# *N*,*N*′-Bis[1-(pyridin-2-yl)­ethyl­idene]benzene-1,4-diamine

**DOI:** 10.1107/S1600536812025834

**Published:** 2012-06-13

**Authors:** Wei Zhou, Rui-Qing Fan, Ping Wang, Yu-Lin Yang

**Affiliations:** a Department of Chemistry, Harbin Institute of Technology, Harbin 150001, People’s Republic of China

## Abstract

In the title compound, C_20_H_18_N_4_, the benzene ring lies about an inversion center. The central benzene-1,4-diamine unit is connected to two pyridine rings by the C=N imine bonds. The dihedral angle between the benzene and pyridine rings is 82.9 (1)°.

## Related literature

For background information on Schiff bases derived from pyridine­carbaldehydes, see: Marjani *et al.* (2009 ▶). For pyridine-derived Schiff bases as bidentate chelating ligands towards metal centers, see: Wu *et al.* (2006 ▶). For a related structure, see: Marjani *et al.* (2011 ▶). For the synthesis of the title compound, see: Yoshida *et al.* (2000 ▶).
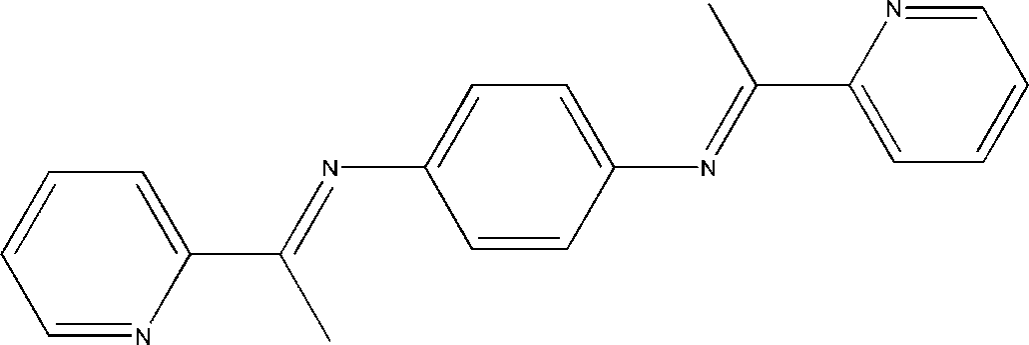


## Experimental

### 

#### Crystal data

C_20_H_18_N_4_
*M* *_r_* = 314.38
Monoclinic, 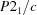

*a* = 5.4660 (11) Å
*b* = 6.8510 (14) Å
*c* = 22.704 (5) Å
β = 90.45 (3)°
*V* = 850.2 (3) Å^3^
*Z* = 2
Mo *K*α radiation
μ = 0.08 mm^−1^
*T* = 293 K
0.50 × 0.48 × 0.19 mm

#### Data collection

Bruker SMART APEX CCD area-detector diffractometer
Absorption correction: multi-scan (*SADABS*; Bruker, 2000 ▶) *T*_min_ = 0.964, *T*_max_ = 0.986
7961 measured reflections
1949 independent reflections
1180 reflections with *I* > 2σ(*I*)
*R*_int_ = 0.037

#### Refinement

*R*[*F* ^2^ > 2σ(*F* ^2^)] = 0.052
*wR*(*F* ^2^) = 0.155
*S* = 1.05
1949 reflections
109 parameters
H-atom parameters constrained
Δρ_max_ = 0.18 e Å^−3^
Δρ_min_ = −0.15 e Å^−3^

### 

Data collection: *SMART* (Bruker, 2000 ▶); cell refinement: *SAINT* (Bruker, 2000 ▶); data reduction: *SAINT*; program(s) used to solve structure: *SHELXS97* (Sheldrick, 2008 ▶); program(s) used to refine structure: *SHELXL97* (Sheldrick, 2008 ▶); molecular graphics: *SHELXTL* (Sheldrick, 2008 ▶); software used to prepare material for publication: *SHELXTL*.

## Supplementary Material

Crystal structure: contains datablock(s) global, I. DOI: 10.1107/S1600536812025834/pv2550sup1.cif

Structure factors: contains datablock(s) I. DOI: 10.1107/S1600536812025834/pv2550Isup2.hkl

Supplementary material file. DOI: 10.1107/S1600536812025834/pv2550Isup3.cml

Additional supplementary materials:  crystallographic information; 3D view; checkCIF report

## Figures and Tables

**Fig. 1.**
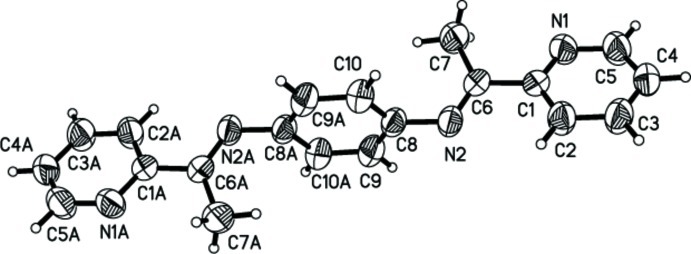
The molecular structure of the title compound with the atom numbering scheme. Displacement ellipsoids are drawn at the 50% probability level. H atoms are presented as small spheres of arbitrary radius.

**Fig. 2.**
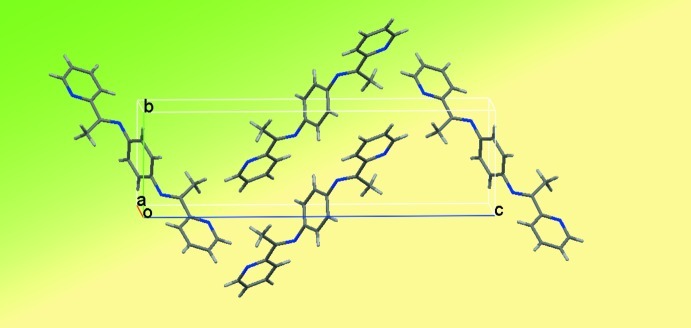
A view of the unit cell packing of the title compound along the a-axis.

## supplementary crystallographic information

### Comment

Schiff bases derived from pyridinecarbaldehydes have received considerable interest in synthetic chemistry (Marjani *et al.*, 2009). *N*,*N*'-bis(1-pyridin-2-ylmethylene)benzene-1,4-diamine is a pyridine derived Schiff base, which acts as bidentate chelating ligand towards metal centers (Wu *et al.*, 2006). It is still challenging to design and rationally synthesize ligands with unique structures and functions. In this regard, we have synthesized the title compound and report its crystal structure in this paper.

The title compound (Fig. 1) lies on an inversion center. The dihedral angle between 1,4-diamine-substituted benzene ring and the pyridine ring is 82.9 (1)°. The bond lengths and bond angles in the title molecule agree very well with the corresponding bond distances and bond angles reported in a closely related compound (Marjani *et al.*, 2011).

### Experimental

The title compound was synthesized by usual Schiff-base condensation of benzene-1,4-diamine and 2-acetyl pyridine. 2-Acetylpyridine (4.50 ml, 0.04 mol) was added in an ethanol (100 mL) solution of benzene-1,4-diamine (2.16 g, 0.02 mol) at room temperature. After the addition was completed, the reaction mixture was heated to 343–353 K and refluxed for 6 h (Yoshida *et al.*, 2000). Then the resultant precipitate was filtered off, washed with ethanol, dried in air and 5.06 g (Yield: 80.6%) brown product was obtained. The crystals of the title compound suitable for X-ray analysis ewere obtained by recrystallization from a mixture of hexane and dichloromethane (3:1).

### Refinement

The C-bound H atoms were positioned geometrically with C—H = 0.93 and 0.96 Å, for aryl and methyl H-atoms, respectively, and allowed to ride on their parent atoms with *U*_iso_(H) = 1.5 *U*_eq_(C-methyl) or 1.2 *U*_eq_(C-aryl).

### Crystal data

**Table:**

C_20_H_18_N_4_	*F*(000) = 332
*M**_r_* = 314.38	*D*_x_ = 1.228 Mg m^−^^3^
Monoclinic, *P*2_1_/*c*	Mo *K*α radiation, λ = 0.71073 Å
Hall symbol: -P 2ybc	Cell parameters from 7961 reflections
*a* = 5.4660 (11) Å	θ = 3.1–27.5°
*b* = 6.8510 (14) Å	µ = 0.08 mm^−^^1^
*c* = 22.704 (5) Å	*T* = 293 K
β = 90.45 (3)°	Block, brown
*V* = 850.2 (3) Å^3^	0.50 × 0.48 × 0.19 mm
*Z* = 2	

### Data collection

**Table:**

Bruker SMART APEX CCD area-detector diffractometer	1949 independent reflections
Radiation source: fine-focus sealed tube	1180 reflections with *I* > 2σ(*I*)
Graphite monochromator	*R*_int_ = 0.037
φ & ω scans	θ_max_ = 27.5°, θ_min_ = 3.1°
Absorption correction: multi-scan (*SADABS*; Bruker, 2000)	*h* = −7→7
*T*_min_ = 0.964, *T*_max_ = 0.986	*k* = −8→8
7961 measured reflections	*l* = −29→29

### Refinement

**Table:**

Refinement on *F*^2^	Primary atom site location: structure-invariant direct methods
Least-squares matrix: full	Secondary atom site location: difference Fourier map
*R*[*F*^2^ > 2σ(*F*^2^)] = 0.052	Hydrogen site location: inferred from neighbouring sites
*wR*(*F*^2^) = 0.155	H-atom parameters constrained
*S* = 1.05	*w* = 1/[σ^2^(*F*_o_^2^) + (0.0759*P*)^2^ + 0.0618*P*] where *P* = (*F*_o_^2^ + 2*F*_c_^2^)/3
1949 reflections	(Δ/σ)_max_ = 0.005
109 parameters	Δρ_max_ = 0.18 e Å^−^^3^
0 restraints	Δρ_min_ = −0.15 e Å^−^^3^

### Special details

**Table:**

Geometry. All e.s.d.'s (except the e.s.d. in the dihedral angle between two l.s. planes) are estimated using the full covariance matrix. The cell e.s.d.'s are taken into account individually in the estimation of e.s.d.'s in distances, angles and torsion angles; correlations between e.s.d.'s in cell parameters are only used when they are defined by crystal symmetry. An approximate (isotropic) treatment of cell e.s.d.'s is used for estimating e.s.d.'s involving l.s. planes.
Refinement. Refinement of *F*^2^ against ALL reflections. The weighted *R*-factor *wR* and goodness of fit *S* are based on *F*^2^, conventional *R*-factors *R* are based on *F*, with *F* set to zero for negative *F*^2^. The threshold expression of *F*^2^ > σ(*F*^2^) is used only for calculating *R*-factors(gt) *etc*. and is not relevant to the choice of reflections for refinement. *R*-factors based on *F*^2^ are statistically about twice as large as those based on *F*, and *R*- factors based on ALL data will be even larger.

### Fractional atomic coordinates and isotropic or equivalent isotropic displacement parameters (Å^2^)

**Table:**

	*x*	*y*	*z*	*U*_iso_*/*U*_eq_	
N1	0.1873 (3)	−0.0704 (3)	0.18884 (6)	0.0716 (5)	
N2	0.2608 (3)	0.1865 (2)	0.05548 (6)	0.0596 (4)	
C1	0.1296 (3)	−0.0097 (2)	0.13471 (7)	0.0494 (4)	
C2	−0.0609 (4)	−0.0910 (3)	0.10286 (8)	0.0648 (5)	
H2B	−0.0943	−0.0491	0.0647	0.078*	
C3	−0.2011 (4)	−0.2348 (3)	0.12820 (9)	0.0726 (6)	
H3A	−0.3327	−0.2888	0.1077	0.087*	
C4	−0.1442 (4)	−0.2972 (3)	0.18377 (8)	0.0688 (6)	
H4A	−0.2354	−0.3943	0.2020	0.083*	
C5	0.0496 (5)	−0.2129 (3)	0.21166 (8)	0.0812 (7)	
H5A	0.0895	−0.2573	0.2492	0.097*	
C6	0.2806 (3)	0.1525 (2)	0.11021 (7)	0.0520 (4)	
C7	0.4428 (5)	0.2623 (4)	0.15204 (8)	0.0847 (8)	
H7A	0.5292	0.3620	0.1310	0.127*	
H7B	0.5581	0.1740	0.1698	0.127*	
H7C	0.3451	0.3213	0.1822	0.127*	
C8	0.3870 (4)	0.3453 (2)	0.02905 (7)	0.0543 (5)	
C9	0.5992 (4)	0.3148 (3)	−0.00200 (8)	0.0620 (5)	
H9A	0.6668	0.1904	−0.0038	0.074*	
C10	0.2881 (4)	0.5319 (3)	0.03045 (8)	0.0620 (5)	
H10A	0.1438	0.5539	0.0508	0.074*	

### Atomic displacement parameters (Å^2^)

**Table:**

	*U*^11^	*U*^22^	*U*^33^	*U*^12^	*U*^13^	*U*^23^
N1	0.0910 (13)	0.0761 (11)	0.0477 (8)	−0.0290 (10)	−0.0058 (8)	0.0046 (8)
N2	0.0716 (11)	0.0492 (8)	0.0578 (9)	−0.0189 (7)	−0.0079 (7)	0.0080 (7)
C1	0.0578 (10)	0.0432 (9)	0.0473 (9)	−0.0031 (7)	0.0024 (7)	−0.0036 (7)
C2	0.0734 (13)	0.0631 (11)	0.0576 (10)	−0.0170 (10)	−0.0117 (9)	0.0134 (9)
C3	0.0763 (14)	0.0710 (13)	0.0702 (12)	−0.0295 (11)	−0.0100 (10)	0.0076 (10)
C4	0.0838 (15)	0.0633 (12)	0.0593 (11)	−0.0227 (10)	0.0052 (10)	0.0074 (9)
C5	0.1055 (19)	0.0862 (15)	0.0517 (10)	−0.0361 (14)	−0.0083 (11)	0.0136 (10)
C6	0.0592 (11)	0.0423 (9)	0.0544 (9)	−0.0056 (8)	0.0012 (8)	−0.0049 (7)
C7	0.1068 (19)	0.0858 (15)	0.0614 (12)	−0.0455 (14)	−0.0065 (11)	−0.0030 (11)
C8	0.0627 (12)	0.0460 (9)	0.0540 (9)	−0.0148 (8)	−0.0114 (8)	0.0043 (7)
C9	0.0688 (13)	0.0434 (9)	0.0738 (12)	−0.0035 (9)	−0.0051 (10)	0.0054 (8)
C10	0.0627 (12)	0.0530 (11)	0.0704 (11)	−0.0102 (9)	0.0026 (9)	0.0048 (9)

### Geometric parameters (Å, º)

**Table:**

N1—C1	1.333 (2)	C5—H5A	0.9300
N1—C5	1.339 (3)	C6—C7	1.497 (3)
N2—C6	1.268 (2)	C7—H7A	0.9600
N2—C8	1.424 (2)	C7—H7B	0.9600
C1—C2	1.381 (3)	C7—H7C	0.9600
C1—C6	1.494 (2)	C8—C9	1.378 (3)
C2—C3	1.377 (3)	C8—C10	1.389 (3)
C2—H2B	0.9300	C9—C10^i^	1.381 (3)
C3—C4	1.366 (3)	C9—H9A	0.9300
C3—H3A	0.9300	C10—C9^i^	1.381 (3)
C4—C5	1.359 (3)	C10—H10A	0.9300
C4—H4A	0.9300		
			
C1—N1—C5	117.03 (16)	N2—C6—C7	125.11 (16)
C6—N2—C8	121.03 (14)	C1—C6—C7	117.61 (14)
N1—C1—C2	121.94 (16)	C6—C7—H7A	109.5
N1—C1—C6	116.62 (15)	C6—C7—H7B	109.5
C2—C1—C6	121.44 (15)	H7A—C7—H7B	109.5
C3—C2—C1	119.34 (16)	C6—C7—H7C	109.5
C3—C2—H2B	120.3	H7A—C7—H7C	109.5
C1—C2—H2B	120.3	H7B—C7—H7C	109.5
C4—C3—C2	119.11 (18)	C9—C8—C10	118.74 (17)
C4—C3—H3A	120.4	C9—C8—N2	120.83 (17)
C2—C3—H3A	120.4	C10—C8—N2	120.26 (18)
C5—C4—C3	117.95 (18)	C8—C9—C10^i^	120.31 (17)
C5—C4—H4A	121.0	C8—C9—H9A	119.8
C3—C4—H4A	121.0	C10^i^—C9—H9A	119.8
N1—C5—C4	124.60 (18)	C9^i^—C10—C8	120.95 (19)
N1—C5—H5A	117.7	C9^i^—C10—H10A	119.5
C4—C5—H5A	117.7	C8—C10—H10A	119.5
N2—C6—C1	117.28 (15)		

Symmetry code: (i) −*x*+1, −*y*+1, −*z*.
